# Intraovarian injection of mesenchymal stem cells improves oocyte yield and *in vitro* embryo production in a bovine model of fertility loss

**DOI:** 10.1038/s41598-020-64810-x

**Published:** 2020-05-15

**Authors:** Patricia F. Malard, Mauricio A. S. Peixer, Joao G. Grazia, Hilana dos Santos Sena Brunel, Luiz F. Feres, Carla L. Villarroel, Luiz G. B. Siqueira, Margot A. N. Dode, Robert Pogue, Joao Henrique M. Viana, Juliana L. Carvalho

**Affiliations:** 1 Universidade Católica de Brasília, Brasília, DF 70790-160 Brazil; 2 Bio Biotecnologia da Reprodução Animal, Brasília, DF 71735-505 Brazil; 3 Cenatte Embrioes, Pedro Leopoldo, MG 33600-000 Brazil; 4 Universidade de Alfenas, Alfenas, MG 37132-440 Brazil; 5 Empresa Brasileira de Pesquisa Agropecuária - EMBRAPA Gado de Leite, Juiz de Fora, MG 36038-330 Brazil; 6 Empresa Brasileira de Pesquisa Agropecuária - EMBRAPA Recursos Genéticos e Biotecnologia, Brasília, DF 70770-917 Brazil; 7 Universidade de Brasília, Brasília, DF 70910-900 Brazil

**Keywords:** Mesenchymal stem cells, Animal biotechnology

## Abstract

Valuable female cattle are continuously subject to follicular puncture (ovum pick-up - OPU). This technique is commonly used for *in-vitro* embryo production, but may result in ovarian lesion. Mesenchymal stem cells (MSC) ameliorate the function of injured tissues, but their use to treat ovarian lesions in cattle has not been established. We investigated whether a local injection of MSC would reduce the negative effects of repeated OPU under acute and chronic scenarios in bovines. First, we performed four OPU sessions and injected 2.5 × 10^6^ MSCs immediately after the 4th OPU procedure (n = 5). The treated organs (right ovary) were compared to their saline-treated counterparts (left), and presented superior production of oocytes and embryos in the three following OPU sessions (P < 0.05). Then, cows with progressive fertility loss went through three OPU sessions. Animals received MSC, saline, or MSC + FSH in both ovaries after the first OPU. In the two following OPU sessions, the MSC and MSC + FSH - treated groups failed to present any significant alteration in the number of oocytes and embryos compared to saline-treated animals. Thus, MSC have beneficial effects on the fertility of OPU-lesioned cows, but not in cows with cystic ovarian disease and chronic ovarian lesions.

## Introduction

The use of *in vitro* embryo technologies has grown worldwide over the past decades and, according to the International Embryo Technology Society (IETS), in 2017 more bovine embryos were produced *in vitro* than generated *in vivo*^1^. The development of transvaginal ultrasound-guided follicle aspiration (a.k.a. ovum pick-up or OPU) and subsequent adaptation for use in cattle^2^ was a key step for the development of *in vitro* embryo production (IVEP) procedures. This technique allowed the repeated recovery of cumulus-oocyte complexes (COC) from live donors, and genetically superior donors could be used for large-scale embryo production, boosting animal breeding programs^3^. The association of OPU and IVEP was successfully used to produce offspring from donors that failed to respond to exogenous FSH treatment^4^, pregnant cows^5^, prepubertal heifers, and young calves^5,6^. Because of its advantages, over 95% of all embryos produced *in vitro* are derived from COC recovered by OPU^1^.

When compared to the previous alternatives to recover COC from live animals, such as laparotomy or laparoscopy, OPU is less traumatic^7^, and can be performed repeatedly in the same donor^7,8^. Thus, this technique is generally considered a safe way to recover COC from cattle^7–9^. However, as with any other needle-based biopsy system, OPU inevitably causes trauma to the ovarian tissue. Ovarian damage subsequent to OPU has been largely neglected, partially due to the internal (non-apparent) nature of the lesions, and also the high regenerative capacity of the ovary, an organ where tissue remodeling occurs naturally over the cycles. Few studies have evaluated ovarian damage caused by OPU, and these reported abnormalities such as tunica albuginea thickening, inflammation, stromal fibrosis, and adhesions^10–12^. Repeated OPU procedures have also been associated with endocrine abnormalities including increased plasma FSH/LH concentrations, follicle growth rate, and incidence of codominance^13^, and it is also a risk factor for the development of cystic ovarian disease^14^.

The cumulative damage caused by successive OPU procedures is associated with a progressive decrease in COC yield and embryo production in donors undergoing repeated collections over several years. The decreased rate, however, is highly variable among donors because it depends on a range of factors including the skill of the technician, type and caliber of the needle used, and aspiration interval. Additionally, there is a significant variation in antral follicle count (AFC) among individuals, as well as among cattle breeds^7,8^. In general, *Bos indicus* breeds have a greater AFC compared with *Bos taurus*, and thus yield more oocytes^15^. OPU sessions yielding over 100 oocytes are not unusual and a record of 564 oocytes collected in a single OPU session from a Nelore donor has been reported^16^. Concomitantly, an OPU-related decrease in the number of COC retrieved was observed in *Bos indicus*, but not in *Bos taurus* and, within the same breed, for cows with high AFC, but not for those with low AFC. Although there are no clear statistics in this regard, field practitioners also report an increasing number of donors, some of them high-value animals, which have compromised reproductive function and failed to produce viable oocytes following repeated OPU cycles (Viana *et al*. personal communication^17^).

Treatment with mesenchymal stem cells (MSC) has been shown to be a valuable therapy in a number of conditions associated with acute and chronic inflammatory processes^18–21^. Stem cells produce a number of cytokines and other soluble mediators that have the ability to modulate the inflammatory and immune activation processes, and potentially minimize inflammation-associated tissue damage^22–24^. In donors undergoing repeated OPU, MSC therapy could be an effective strategy to reduce the detrimental effects of inflammation and subsequent fibrosis on ovarian function. Stem-cell therapy has been shown to improve ovarian function in laboratory animal models in which infertility was artificially induced^25–29^. However, the clinical use in large farm animals is still under discussion^30,31^. The present study was designed to evaluate the effect of intraovarian MSC treatment on oocyte yield and embryo production. We hypothesized that MSC treatment would reduce the negative effects of repeated OPU on donor performance, by improving oocyte quantity and/or quality. In this regard, we designed two experiments, aiming to evaluate the effects of intraovarian treatment with MSC in ovaries under acute and chronic injury processes due to follicular puncture.

## Results

### MSC treatment promotes higher production of oocytes, embryos and expanded blastocysts in acute OPU-induced ovarian lesions

**Figure 1:**
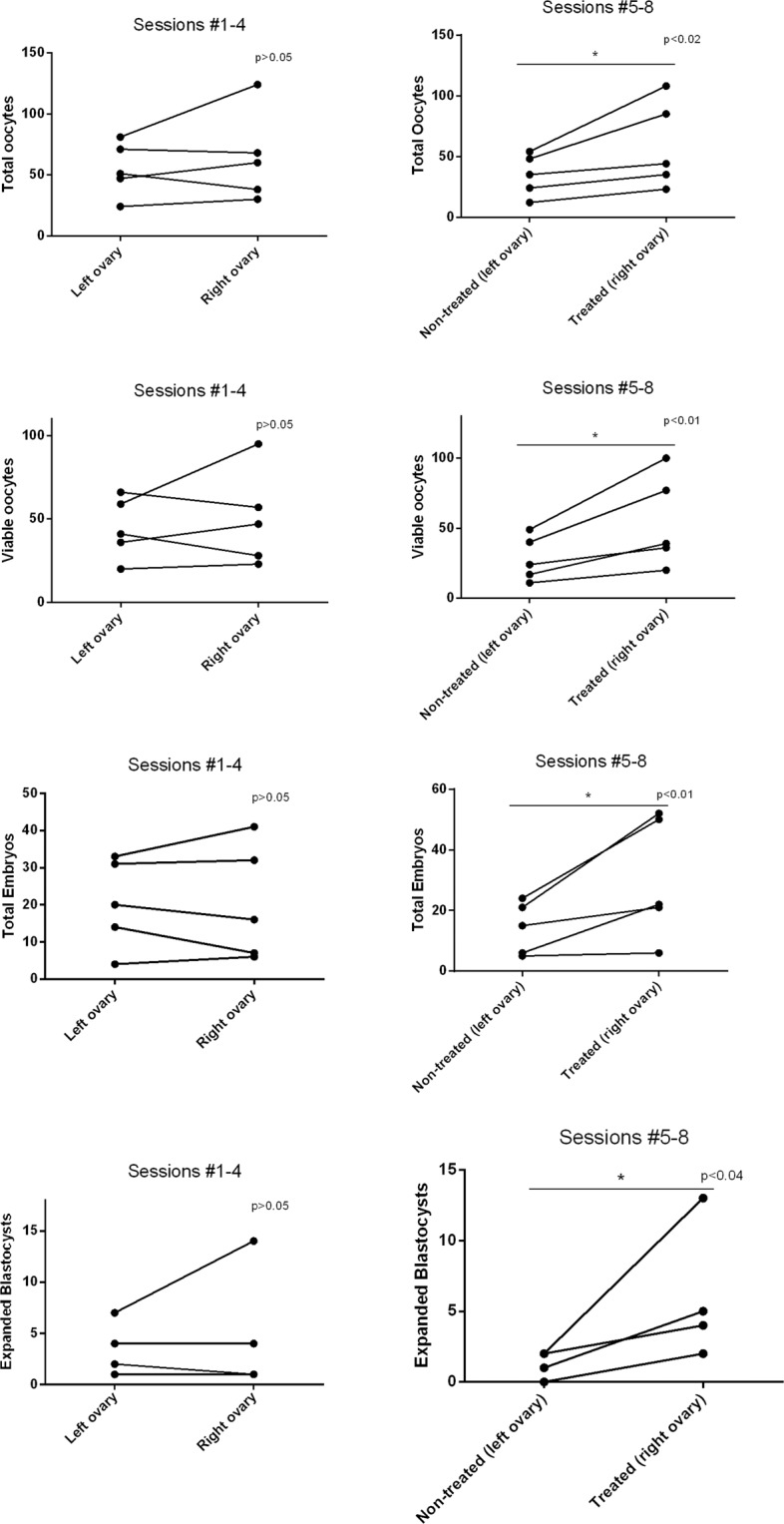
Effect of intraovarian injection of mesenchymal stem cells (MSC) on total OPU/IVEP acute outcomes. Treatments were performed immediately after the 4th OPU/IVEP session. The right ovary received MSC treatment, while the left ovary remained as a control and received DMPBS (n = 5 per group). Endpoints are shown according to the ovary, before (sessions 1 to 4) and after (sessions 5 to 8) treatment. *P < 0.05.

The animals presented no difference (P > 0.05) in any of the analyzed parameters between the ovaries (right *versus* left) before MSC treatment (OPU sessions 1 to 4). Thus, it is possible to attribute any differences between treated (right) and untreated (left) ovaries in OPU sessions 5 to 8, to MSC infusion. Treated ovaries presented more total (P < 0.02) and viable oocytes (P < 0.01), when compared to the untreated ovaries, resulting in more embryos produced *in vitro* (P < 0.01), as well as superior production of early and expanded blastocysts (Fig. 1, Table 1).

**Table 1:** Average results of ovum pick-up and *in vitro* embryo production according to ovary, before (sessions 1 to 4) and after (sessions 5 to 8) injection of mesenchymal stem cells in the right ovaries and DMPBS in the left ovaries.

Endpoint	Right ovary	Left ovary	P value	Right ovary (treatment)	Left ovary (control)	P value
	Sessions #1 to 4			Sessions #5 to 8		
Total oocytes	15.9 ± 2.6	13.7 ± 1.6	0.48	15.3 ± 2.2	8.7 ± 1.2	**0.02**
Viable oocytes	12.4 ± 1.9	11.1 ± 1.4	0.60	13.6 ± 2.1	7.1 ± 1.0	**0.01**
Total Embryos	5.1 ± 1.0	5.2 ± 1.1	0.94	7.6 ± 1.2	3.6 ± 0.6	**0.01**
Expanded blastocysts	2.8 ± 0.6	3.0 ± 0.8	0.83	4.4 ± 0.9	2.1 ± 0.4	**0.03**
Blastocysts	1.3 ± 0.4	1.4 ± 0.4	0.75	1.8 ± 0.4	1.2 ± 0.3	0.12
Early blastocysts	1.1 ± 0.4	0.8 ± 0.3	0.61	1.4 ± 0.3	0.4 ± 0.1	**0.04**

**Figure 2:**
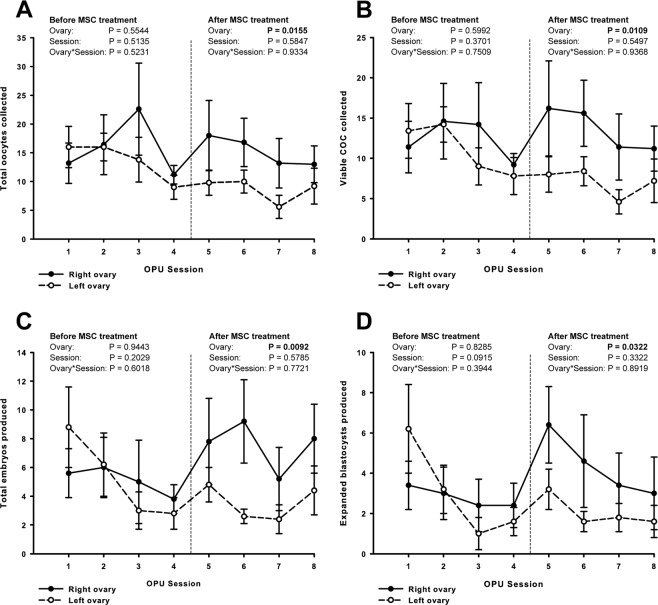
Effect of intraovarian injection of mesenchymal stem cells (MSC) on OPU/IVEP acute outcomes per session. Treatments were performed immediately after the 4th OPU/IVEP session. The right ovary received MSC treatment, while the left ovary remained as a control and received DMPBS. Endpoints are shown as mean ± SEM (n = 5 per group) according to the ovary, before (sessions 1 to 4) and after (sessions 5 to 8) treatment. (**A**) total number of oocytes collected; (**B**) number of viable COC; (**C**) total number of embryos produced; (**D**) number of expanded blastocysts produced.

OPU session did not present any effect on the number of total or viable oocytes recovered, nor on the number of total or expanded blastocysts produced in any of the experimental groups (Fig. 2A to D). The same lack of effect was observed for the ovary x OPU session interaction. The percentage of viable oocytes recovered was higher in treated ovaries compared to the untreated counterparts (89.1% vs 81.5%, P < 0.05). However, blastocyst rates did not differ between treated and untreated ovaries before or after treatment (50.4% vs 55.5%, P > 0.05).

**Figure 3:**
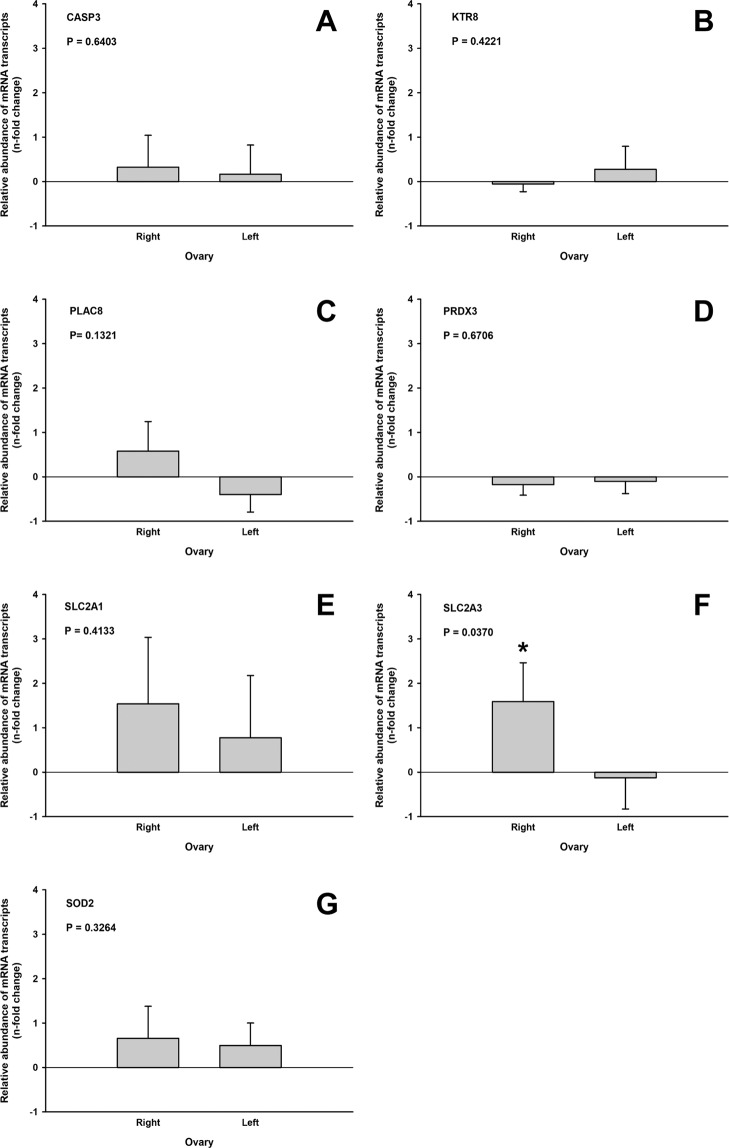
Gene expression patterns in blastocysts produced *in vitro* from oocytes recovered after intraovarian treatment with MSC (right ovary) or from untreated ovaries (left ovary) after acute lesion. Gene expression in embryos from slaughterhouse ovaries was used as a reference (expression value = 0). Graphs show the relative abundance of mRNA of CASP3 (**A**), KRT8 (**B**), PLAC8 (**C**), PRDX3 (**D**), SLC2A1 (**E**), SLC2A3 (**F**), and SOD2 (**G**) genes determined by qPCR. Data were normalized by ΔΔC_t_, using Glyceraldehyde-3-phosphate dehydrogenase (GAPDH) and β-Actin (ACTB) as endogenous controls. * Indicates statistical difference (P < 0.05).

There was no difference (P > 0.05) in the abundance of transcripts of KTR8, PLAC8, SLC2A1, CASP3, PROX3, or SOD2 in the embryos produced from treated and untreated ovaries, or from slaughterhouse ovaries. However, SLC2A3 was overexpressed (P = 0.04) after MSC treatment, compared with controls (Fig. 3).

### MSC treatment doesn’t significantly alter the production of oocytes, embryos and expanded blastocysts in chronic OPU-induced lesion ovaries

**Figure 4:**
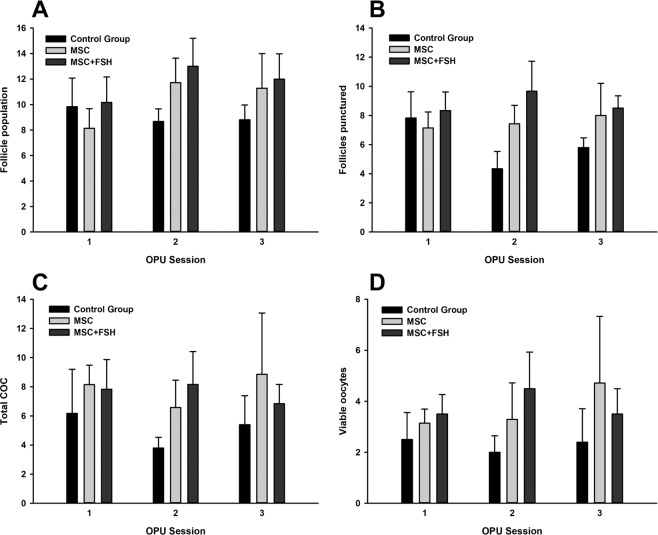
Ovarian and OPU outcomes in Gir cows with low IVEP records associated with their use as oocyte donors for long periods (chronic lesion). Cows received DMPBS (control group), mesenchymal stem cells (MSC group), or mesenchymal stem cells followed by FSH priming (MSC + FSH group). There was no statistical difference among groups (P > 0.05).

Active immunization against GnRH was effective to reduce the number of large follicles present in the ovaries (from 2.6 ± 0.3 to 0.2 ± 0.1, P < 0.05 - Data not shown). Only one cow still presented a large ovarian cyst (33 mm diameter) and was removed from the experiment. In the remaining 18 cows, the average AFC after immunization was 11.2 ± 0.8 (range 3 to 19), and there was no difference among experimental groups (11.2 ± 1.4, 11.1 ± 2.0, 11.4 ± 1.3 for Control group, MSC and MSC + FSH, respectively, P > 0.05), as shown in Fig. 4, Figure S1 and Table 2. One animal from the control group died before OPU session 3.

**Table 2:** Effect of treatment with mesenchymal stem cells (MSC) or MSC associated with FSH on the number of matured COC, cleaved embryos and day-7 blastocysts in Gir (*Bos indicus*) cows that previously underwent multiple OPU sessions and had a history of low embryo production.

Endpoint	Group	OPU session		
		1	2	3
COC matured				
	Control	2.5 ± 1.1	2.0 ± 0.6	2.4 ± 1.3
	MSC	3.1 ± 0.6	3.3 ± 1.4	4.7 ± 2.6
	MSC + FSH	3.5 ± 0.8	4.5 ± 1.4	3.5 ± 1.0
Cleaved				
	Control	2.3 ± 1.1	1.6 ± 0.5	2.2 ± 1.1
	MSC	2.3 ± 0.5	2.3 ± 0.9	4.0 ± 1.6
	MSC + FSH	2.5 ± 0.5	3.5 ± 1.3	3.0 ± 0.7
Blastocysts				
	Control	0.2 ± 0.2	0.4 ± 0.4	0.2 ± 0.2
	MSC	0.3 ± 0.3	0.0 ± 0.0	0.2 ± 0.2
	MSC + FSH	0.3 ± 0.2	0.2 ± 0.2	0.0 ± 0.0

The OPU outcomes are shown in Fig. 4(A–D), Figure S1 and Table 2. Despite individual variation in response to the MSC and MSC + FSH treatments, there was no difference (P > 0.05) among groups in AFC, number of follicles aspirated, retrieved oocytes, or viable oocytes. Due to the low number of viable oocytes, the numbers of cleaved embryos and blastocysts were low and did not differ among groups (Table 2, P > 0.05). Over the same period, blastocyst rates of the IVEP laboratory using the same maturation, fertilization, and culture media and conditions, and with the same breeds (Gir x Holstein), ranged from 16.4 to 27.0%.

## Discussion

Mesenchymal stem cells have shown therapeutic efficacy for different purposes, such as immunological disorders^32^, variable chronic lesions^33^, and ischemia^34^. In the context of ovarian physiology, MSC were shown to be beneficial in the treatment of different disorders. Even though to date, most studies have been performed in rodents, MSC have promoted positive effects on chemotherapy-induced lesions, premature ovarian failure, and polycystic ovary syndrome^35–39^. The positive results can be explained, at least in part, by the effects of MSC on granulosa cells, as well as their anti-inflammatory, antiapoptotic, and regenerative effects, and potential paracrine actions^40^. Despite the positive results obtained, rodents differ significantly from humans when it comes to the female reproductive system^41^. In this sense, not only are cattle more similar to humans in the context of female tract physiology, but they also constitute an economically relevant model which may positively impact the economy by presenting longer reproductive life after MSC therapy.

The present study evaluated, for the first time, the use of intraovarian MSC as a treatment to reduce the negative effects of repeated OPU in donor cattle. In Experiment 1 we first tested the MSC in cows that underwent OPU for a short period (four sessions), thus reflecting the acute effects of puncture injuries. Our results demonstrated that the treated ovaries yielded more total and viable oocytes, leading to an increase in embryo production per OPU session. We then tested in Experiment 2 whether the MSC treatment would also bring any benefit to cows with a history of multiple OPU sessions and progressive failure in producing embryos and pregnancies. In spite of a positive variation in AFC and oocyte recovery in the treated cows, there was no significant difference compared with controls.

Stem cells have a positive tropism to any area undergoing an inflammatory process. Thus, to ensure cells would act specifically within the ovary, we used an intraovarian injection, a non-usual treatment route in large animal veterinary practice. A number of studies reported intrafollicular^42–44^, intraluteal^45,46^, or intraovarian^44,47^ administration of drugs, but most, if not all, were only for experimental purposes. As the aim of the present study was to test an alternative for the treatment of oocyte donors used for commercial IVEP, a previous study was performed to test the safety of this procedure and validate the amount of MSCs injected (Peixer *et al*. in press). The experiment was performed by injecting MSC into healthy cow ovaries and no adverse events were registered, confirming the safety of the procedure. In Experiment 1, we performed additional perforations in the ovaries before injection of saline or MSC, to reduce potential differences in inflammation between ovaries from which a greater or smaller number of oocytes was recovered.

An important potential source of variation in our results was the predictable differences in AFC among donors^8^. To account for this, in Experiment 1 we allocated the right and left ovaries of each animal to treatment and control groups, respectively. The lack of difference in any endpoint between the ovaries before treatment demonstrates that we succeeded in balancing individual variations, and differences after the fifth OPU sessions are likely to be a result of MSC treatment. In our experimental model we cannot ensure that MSC would not migrate from one ovary to the other. However, during migration throughout the circulatory system, these cells could be equally attracted to any other area with inflammation (e.g., other areas perforated during OPU such as the vaginal wall), and the number of cells eventually reaching the contralateral ovary is probably negligible.

In Experiment 2, most donors had varied records of chronic COD and refractoriness to conventional GnRH or P4 treatments, so they were treated with an anti-GnRH vaccine. Previous studies have shown that cows receiving either GnRH immunization or GnRH agonist, despite having suppressed follicle growth, can be used as oocyte donors and produce embryos *in vitro*^14,48^. Therefore, we decided to include a group with FSH priming, and thus we could not use the same experimental model as in Experiment 1. To minimize the potential effect of differences in AFC among donors, the cows were balanced-distributed according to AFC into the experimental groups.

In Experiment 1 (animals with acute lesions), the MSC treatment positively affected the number of total and viable oocytes, as well as the proportion of viable oocytes, suggesting a beneficial effect on follicle development. This positive effect could be due to either the increased follicle recruitment or a reduced follicle atresia. In the current study, differences were observed shortly after treatment, suggesting that the MSC had a positive effect on the population of growing antral follicles that were aspirated within a few weeks after treatment, and thus were recruited from the pool of primordial follicles before MSC treatment. This observation is in line with the anti-apoptotic effect and subsequent increase in antral follicle population observed after this type of cell therapy in other species^28,29^.

The inflammation associated with repeated OPU procedures may cause the presence of high concentrations of inflammatory signals in the ovary. Infections in other organs such as the uterus or mammary gland have also been associated with an increase in fibrotic tissue in the ovary, disturbed folliculogenesis, abnormal intrafollicular environment, altered gene expression in granulosa cells, and reduced oocyte levels of GDF9 and developmental potential^49–52^. The presence of bacterial LPS induces the accumulation of inflammatory mediators such as IL-1beta, IL-6, and IL-8^50,53,54^, which are also likely to be increased due to the mechanical damage caused by OPU.

We can infer that the presence of MSC may have negatively modulated the production of inflammation-related cytokines, as observed in mice^27^, and thus minimized their detrimental effects on growing follicles, reducing atresia and improving oocyte quality. Concordantly, the increase observed in IVEP outcomes was due to the recovery of a greater number of viable COC.

The present data support the hypothesis that differences in oocyte quality may impact embryo development. We are not sure why among the genes evaluated, only SLC2A3 was overexpressed in embryos produced from the treated ovaries, compared with untreated ovaries or slaughterhouse controls. However, this result suggests that metabolism and glucose uptake by the embryo may have been affected by MSC treatment. Glucose is the main energy substrate for the embryo; however, the embryo’s glycolytic ability is low initially, but increases markedly at the blastocyst stage^55^. A positive correlation was observed between glucose uptake and viability of preimplantation embryos^56^. Since glucose is hydrophilic, it must be transported into the cell by transporters known as the solute carrier family 2 (SLC2A), among which SLC2A3 is particularly important for embryos. A study in mice reported that when the *SLC2A3* gene was deleted, embryo development until the blastocyst stage was not affected, but embryo lethality was observed shortly after implantation^57^.

MSC treatment was not effective to increase IVEP in Experiment 2. In this case, we can speculate that the chronic inflammatory process in the ovaries due to repeated OPU over the years may have compromised follicular population or ovarian physiology in a way that could no longer be restored by MSC. For instance, repeated OPU causes accumulation of scar tissue in ovarian stroma^12^, resulting in ovary hardening, as clinically observed^11^. The changes observed in ovarian stiffness could affect preantral follicle development, as observed when preantral follicles are cultured in alginate hydrogel with different densities^58^, and result in a progressive reduction in AFC, as observed in the cows enrolled in Experiment 2. In fact, the average AFC values for these cows were about one third of those observed in healthy heifers of the same herd (11.2 ± 0.8 vs. 30.8 ± 1.1, respectively). Moreover, it is likely that there was also an interplay among the chronic ovarian inflammation, endocrine disbalance, and aging, which may have contributed to the very low IVEP outcome of most of these cows.

In summary, the present study presents the first evidence that the intraovarian injection of MSC has beneficial effects on AFC and on IVEP in zebu cows undergoing repeated OPU. However, such effects may be limited depending on how long cows have been used as oocyte donors, reflecting the extent of the damage in the ovarian tissue.

## Methods

Unless otherwise indicated, all reagents were purchased from Sigma-Aldrich (St. Louis, MO, USA).

### Animals and location

Multiparous, lactating Nelore cows at 60d after parturition and non-lactating Gir oocyte donors (N = 5 and N = 19, respectively, both *Bos taurus indicus* breeds), were used. These animals were from two commercial farms, one at Flores de Goiás, GO, and the other at Leopoldina, MG, Brazil. The cows were raised on pasture (mostly *Brachiaria decumbens*), with *ad-libitum* access to water and to vitamin and mineral supplements throughout the experiment. Nelore cows were selected based on reproductive soundness, inferred by the lack of pathological conditions in the genital tract, as determined by ultrasound scanning and rectal palpation. On the other hand, Gir cows were previously submitted to multiple sessions of OPU and were to be culled due to low performance on IVEP. According to their records, in the last fifteen OPU-IVEP sessions these cows had a drop of 47.8% in the number of viable oocytes recovered and of 77.5% in the number of embryos produced. When first examined for this study, all Gir cows presented chronic cystic ovarian disease (COD), with an average of 2.6 ± 0.3 follicles above the expected diameter of the ovulatory follicle in Gir (circa 12 mm)^13^, and 9 out of 19 also presented mucometra. These cows were 10.4 ± 0.5 years old when this study was performed.

This study was approved by the Committee for Ethics in the Use of Animals of the Universidade Católica de Brasília (CEUA-UCB, protocol 003/18), and all experiments were performed in accordance with relevant guidelines and regulations.

### Experimental design

In experiment 1, we evaluated the effect of intraovarian injection of MSC on oocyte yield, quality, and developmental potential during *in vitro* embryo production. To account for the expected variability in AFC, all cows (Nelore, N = 5) were treated in one ovary, while the other was used as a control. Each cow underwent eight OPU sessions at 15-day intervals. Immediately after the fourth OPU session, an additional lesion was induced in each ovary by 30 punctures, performed with a 16 G Jelco needle, to ensure that substantial acute injury would be present, regardless of the number of punctures needed for follicle aspiration in each cow. Six hours later, the left ovary received an injection of 500 μL Dulbecco’s Modified Phosphate Buffer Saline (DMPBS) (control ovary), whereas the right ovary received 500 μL DMPBS with 2.5 × 10^6^ allogeneic MSC (treated ovary). In both cases, the final volume was distributed across three points on the ovarian cortex. The results of oocyte yield and embryo production in the four sessions before and in the four sessions after treatment were recorded for each ovary and donor.

In experiment 2, we evaluated the potential benefits of MSC treatment in cows with low IVEP performance associated with long-term effects of OPU. The cows underwent a gynecological exam by B-mode and color Doppler ultrasonography to characterize ovarian status. To control for the potential detrimental effects of COD, cows were treated with two injections of an anti-GnRH vaccine (Bopriva, Zoetis Saúde Animal, São Paulo, Brazil), given 30 days apart, as previously described^14^. Twenty days later, cows were re-evaluated and were distributed in a balanced manner according to ovarian antral follicle count (AFC) into one of the following experimental groups: a) control group (CG), intraovarian injection of saline; b) intraovarian injection of MSC (treatment in both ovaries); or c) injection of MSC after priming with 150 IU FSH (Pluset, Hertape Calier Saúde Animal, Juatuba, Brazil). All cows underwent three OPU sessions, 20 days apart. MSC were injected in groups designated MSC and MSC + FSH, immediately after OPU session 1, and FSH was injected in the MSC + FSH group 48 h before OPU sessions 2 and 3. Oocyte and embryo yield were recorded for each donor and results compared among groups and OPU sessions.

In both experiments, the endpoints evaluated were the number and quality of the oocytes recovered, and number and rates of *in vitro* embryo production. In Experiment 1, the gene expression pattern was evaluated in the blastocysts, as described below.

### Collection and characterization of mesenchymal stem cells

The method used to isolate, culture, and freeze MSC was previously described (Peixer *et al*. in press). Briefly, a sample of adipose tissue was recovered at a slaughterhouse from a healthy bull, washed in saline, treated with hyaluronidase, and filtered. The cells isolated were cultured in Dulbecco´s modified Eagle´s media (DMEM), at 37.5 °C and 5% CO_2_ atmosphere. The medium was renewed at 24 h and non-adherent cells discarded. At 80% confluence, cells were isolated with trypsin, and frozen in liquid N_2_ in a dimethyl sulfoxide (DMSO) solution with fetal calf serum (FCS) at a final concentration of 1 × 10^6^ cells/mL. Characterization of MSC was carried out according to guideline 131 of the International Society for Cellular Therapy^59^. Immunophenotyping was performed by flow cytometry and Amnis image quantification using positive (CD29 (rat anti-human), CD44 (rat anti-equine), and CD90 (goat anti-canine)) and negative (CD34 (rat anti-human)) surface markers. The ability of MSC to differentiate into osteoblasts, chondrocytes and adipocytes was also evaluated, as described previously^60,61^. Samples were also screened for the presence of potential contaminants (bacteria, fungi, and mycoplasma). Cell viability after thawing was evaluated by flow cytometry using annexin-Alexa Fluor 488 and propidium iodide (Thermo-Fisher Scientific, Bremen, Germany).

### Transvaginal guided follicular aspiration and MSC injection

Cumulus-oocyte complexes were recovered by OPU, as described elsewhere^13^. Briefly, all follicles larger than 3 mm were aspirated using a portable ultrasound device (Aloka SSD 500, Aloka Co., Tokyo, Japan; or MyLab 30 Gold, Esaote, Genova, Italy) equipped with a biopsy guide, and disposable 20 G needles (WTA, Cravinhos, Brazil). A vacuum pressure of 80 to 100 mm Hg was used. The follicular fluid was recovered in 50 mL tubes containing 15 mL of DPBS supplemented with 1% FCS and 5 IU/mL sodium heparin (Hemofol, Cristália, São Paulo, Brazil). The recovered COC were classified according to the number of cumulus cell layers and cytoplasm morphology. Only COC classified as viable were transferred to cryotubes (Corning, 1.2 mL, New York, USA) containing maturation medium that consisted of TCM199 (Gibco, New York, USA) supplemented with 0.05 IU/mL FSH (Pluset, Hertape-Calier, Barcelona, Spain), 1 mg/mL estradiol, and 10% FCS, and kept in a portable incubator at 38.5 °C until transportation to the IVEP laboratory. The total number and the grade of oocytes recovered from each ovary (right or left) and from each donor were recorded.

The intraovarian injection of MSC was performed with the same ultrasound device used for OPU. After thawing and washing twice in PBS, the MSC suspension was loaded into a 1 mL syringe, which was connected to a new aspiration line. Ultrasound imaging was used to select three regions of the ovarian cortex free of antral follicles or luteal tissue, and to guide the injection needle. Each region was then injected with approximately 1/3 of the MSC suspension volume.

### *In vitro* embryo production

Mature COC were obtained after a 24 h incubation at 38.5 °C under an atmosphere of 5% CO_2_ in air, using the same media used for transportation. Expanded COC were then washed and transferred to fertilization media consisting of Tyrode’s albumin lactate pyruvate (TALP) supplemented with 10 μg/mL heparin, 20 μM D-penicillamine, 10 μM hypotaurine, and 1 μM epinephrine. For all fertilization procedures, frozen semen samples from a single bull of proven fertility (Aberdeen Angus or Holstein) were used at 1 × 10^6^ spermatozoa/mL. Oocytes and spermatozoa were co-incubated for 20 h at 38.5 °C in a humidified incubator with 5% CO_2_. After coincubation (day 0), presumptive zygotes were transferred to 50 µL drops of synthetic oviduct fluid (SOFaa) supplemented with essential and non-essential amino acids, 0.34 mM sodium tricitrate, 2.77 mM myo-inositol, and 10% FBS under mineral oil. The zygotes were cultured for 7 days, when embryo production, developmental stage and quality were evaluated. The embryos were also evaluated on Day 2 (D2) post-insemination for cleavage and on Day 7 (D7) for blastocyst development.

### Gene Expression Quantification by Real Time Quantitative PCR (RT-qPCR)

For gene expression analysis, the relative abundance of transcripts for eight target genes involved in embryo quality was quantified by RT-qPCR in D7 *in vitro*-produced embryos. The selected genes were: placenta-specific 8 (PLAC8), keratin protein 8 (KRT8), heat stress (heat shock 27-kDa protein 1 (HSPB1)); apoptosis cysteine peptidase 3 (CASP3), superoxide dismutase 2 (SOD2), solute carrier family 2 member 1 (SLC2A1), solute carrier family 2 member 3 (SLC2A3) and Peroxiredoxin 3 (PRDX3). Three pools of 29 to 32 embryos for each treatment group were used. Total RNA was isolated using the RNeasy Plus Micro Kit (Qiagen, Hilden, Germany), according to the manufacturer’s instructions. Complementary DNA synthesis was performed as described in Leme *et al*.^62^. The RT-qPCR reactions were performed using the Fast SYBR Green Master Mix Kit (Applied Biosystems, Foster City, California, USA) in an Applied Biosystem 7500 Fast Real Time PCR System (Applied Biosystem). Each sample was analyzed in triplicates with an amplification efficiency between 90 and 110%, and the specificity of each PCR product was determined by analyzing the melting curve and size of amplicon on an agarose gel. The reactions were performed in a final volume of 25 μL, using cDNA equivalent to 0.8 embryos per reaction. The qPCR conditions were: 95 °C for 5 minutes followed by 50 cycles of denaturation at 95 °C for 10 seconds and then annealing and extension at 60 °C for 30 seconds. The primer names, sequences, fragment sizes and annealing temperatures are listed in Table 3. The average expression level of two constitutive genes, Glyceraldehyde-3-phosphate dehydrogenase (GAPDH) and β-Actin (ACTB), was used for data normalization. The relative quantification of each gene was calculated by the ΔΔCt method with efficiency correction^63^.

**Table 3:** Specific primers used for gene expression analysis by quantitative polymerase chain reaction (qPCR).

Primer	Sequence (5′ – 3′)	Amplicon size (bp)	Primer *concentration* (nM)	GeneBank access number or reference
GAPDH	Forward: GGC GTG AAC CAC GAG AAG TAT AA Reverse: CCC TCC ACG ATG CCA AAG T	118	300	NM_001034034.2
ACTB	Forward: GGC ACC CAG CAC AAT GAA GAT CAA Reverse: ATC GTA CTC CTG CTT GCT GAT CCA	126	300	XM_010845770.1
CASP3	Forward: GCC CAG GAC TTT AGC AGT CA Reverse: AAA TGT GAG CGC CTT TGT T	185	250	NM_001077840.1
SLC2A1	Forward: CAG GAG ATG AAG GAG GAG AGC Reverse: CAC AAA TAG CGA CAC GAC AGT	258	250	NM_174603.3
SLC2A3	Forward: ACT CTT CAC CTG ATT GGC CTT GGA Reverse: GGC CAA TTT CAA AGA AGG CCA CGA	145	300	X12877 (El-Sayed *et al*. 2006)
PLAC8	Forward: GAC TGG CAG ACT GGC ATC TT Reverse: CTC ATG GCG ACA CTT GAT CC	140	300	NM_016619
KRT8	Forward: GGT TCT GGA GAC CAA ATG GAA Reverse: CCG ACG GAG GTT GTT AAT GTA G	97	300	NM_001033610.1
PDRX3	Forward: GGC AGG AAC TTT GAT GAG AT Reverse: GTG TGT AGC GGA GGT ATT TC	205	300	NM_174643.1
SOD2	Forward: TTG CTG GAA GCC ATC AAA CGT GAC Reverse: AAT CTG TAA GCG TCC CTG CTC CTT	135	300	NM_ 201527

### Statistical analysis

First, the Shapiro-Wilk test was used to assess the normality of the data. The data referring to the number of early blastocysts in OPU sessions 1 to 4 and 5 to 8 (experiment 1) were transformed into natural logarithms. The total oocytes recovered in sessions 1 to 4 were transformed into square root. All other endpoints were analyzed using non-transformed data. Analyses were performed to assess the main effects of ovary (treated *versus* untreated), OPU session, as well as their interactions. In order to test for natural differences between right and left ovaries, the data referring to the OPU sessions performed before (1 to 4) treatment were analyzed separately from the data obtained from OPU sessions performed after MSC treatment (5 to 8). Statistical analysis was also performed in order to compare ovaries before and after treatment. In data analyses for MSC treatment during chronic lesion (Experiment 2), the follicle population, the number of follicles aspirated, retrieved COC, and viable oocytes were normally distributed. The main effects of treatment, OPU session, and their interactions were analyzed. The SAS MIXED procedure with a REPEATED statement was used for both experiments, to account for the autocorrelation between sequential measurements (SAS University Edition; SAS Institute Inc., Cary, NC, USA). Whenever a significant main effect of ovary was detected, the Student’s *t* test was used to compare differences among means in Experiment 1. Whenever a significant effect of treatment, session or interaction was observed, the Tukey's post hoc test was used to compare differences among means in Experiment 2. Data are presented as mean ± standard error of the mean (SEM) of non-transformed data. In the acute inflammation experiment (Experiment 1), comparisons of gene expression among groups were performed using ANOVA and Tukey’s test or Kruskal-Wallis and Mann-Whitney tests for normally and non-normally distributed data, respectively. These analyses were performed using the software GraphPad Prism 6, and a P-value <0.05 indicated statistical significance.

## Supplementary information

Supplementary Information.
