# Supplementary material for: Intraovarian injection of mesenchymal stem cells improves oocyte yield and in vitro embryo production in a bovine model of fertility loss
Source: Sci Rep. 2020 May 15;10:8018. doi: 10.1038/s41598-020-64810-x (PMC7229041; doi:10.1038/s41598-020-64810-x)
Supplement: Supplementary file 1 — Supplementary Information. [file 41598_2020_64810_MOESM1_ESM.pdf]

Intraovarian injection of mesenchymal stem cells improves oocyte yield and in vitro embryo production in a bovine model of fertility loss

Article Type: Original Research Article

Patricia F Malard[a,b*], Mauricio A S Peixer[a,b], Joao G Grazia[c], Hilana dos Santos Sena Brunel[b], Luiz F Feres[d], Carla L Villarroel[b], Luiz G B Siqueira[e], Margot A N Dode[f], Robert Pogue[a], Joao Henrique M Viana[f#], Juliana L Carvalho[a,g#*]

[a] Universidade Católica de Brasília, Brasília, DF, 70790-160 Brazil
[b] Bio Biotecnologia da Reprodução Animal, Brasília, DF, 71735-505 Brazil
[c] Cenatte Embrioes, Pedro Leopoldo, MG, 33600-000 Brazil
[d] Universidade de Alfenas, Alfenas, MG, 37132-440 Brazil
[e] Empresa Brasileira de Pesquisa Agropecuária - EMBRAPA Gado de Leite, Juiz de Fora, MG, 36038-330 Brazil
[f] Empresa Brasileira de Pesquisa Agropecuária - EMBRAPA Recursos Genéticos e Biotecnologia, Brasília, DF, 70770-917 Brazil
[g] Universidade de Brasília, Brasília, DF, 70910-900 Brazil

ORCID list:
Patrícia F Malard: https://orcid.org/0000-0002-3073-4748
Maurício A S Peixer: https://orcid.org/0000-0002-9118-7768
João G Grazia: https://orcid.org/0000-0002-9976-681X
Hilana dos Santos Sena Brunel: https://orcid.org/0000-0002-9425-1593
Luis F Feres: http://orcid.org/0000-0001-7566-2859
Carla L Villarroel: https://orcid.org/0000-0002-5125-9798
Luiz G B Siqueira: https://orcid.org/0000-0002-2800-5829
Margot A N Dode: https://orcid.org/0000-0002-1096-0457
Robert Pogue: https://orcid.org/0000-0002-8789-3512
João Henrique M Viana: https://orcid.org/0000-0002-3742-2368
Juliana L Carvalho: https://orcid.org/0000-0002-1423-0523

#Both authors contributed equally to the work and are co-senior authors.

*Corresponding Authors:
Patrícia Furtado Mallard
Address: SMPW, Quadra 05, Conjunto 05, Lote 01, Casa C, Brasília, DF,
Phone: +55(61) 3382-7407
Correspondence to: patricia@biotecnologiaanimal.com.br

*Juliana Lott de Carvalho
Address: SMLN, Condominio Prive I, Quadra 03, Conjunto C15, Brasília, DF,
Phone: +55(61) 99907-7879
Correspondence to: julianalott@gmail.com

Supplementary material

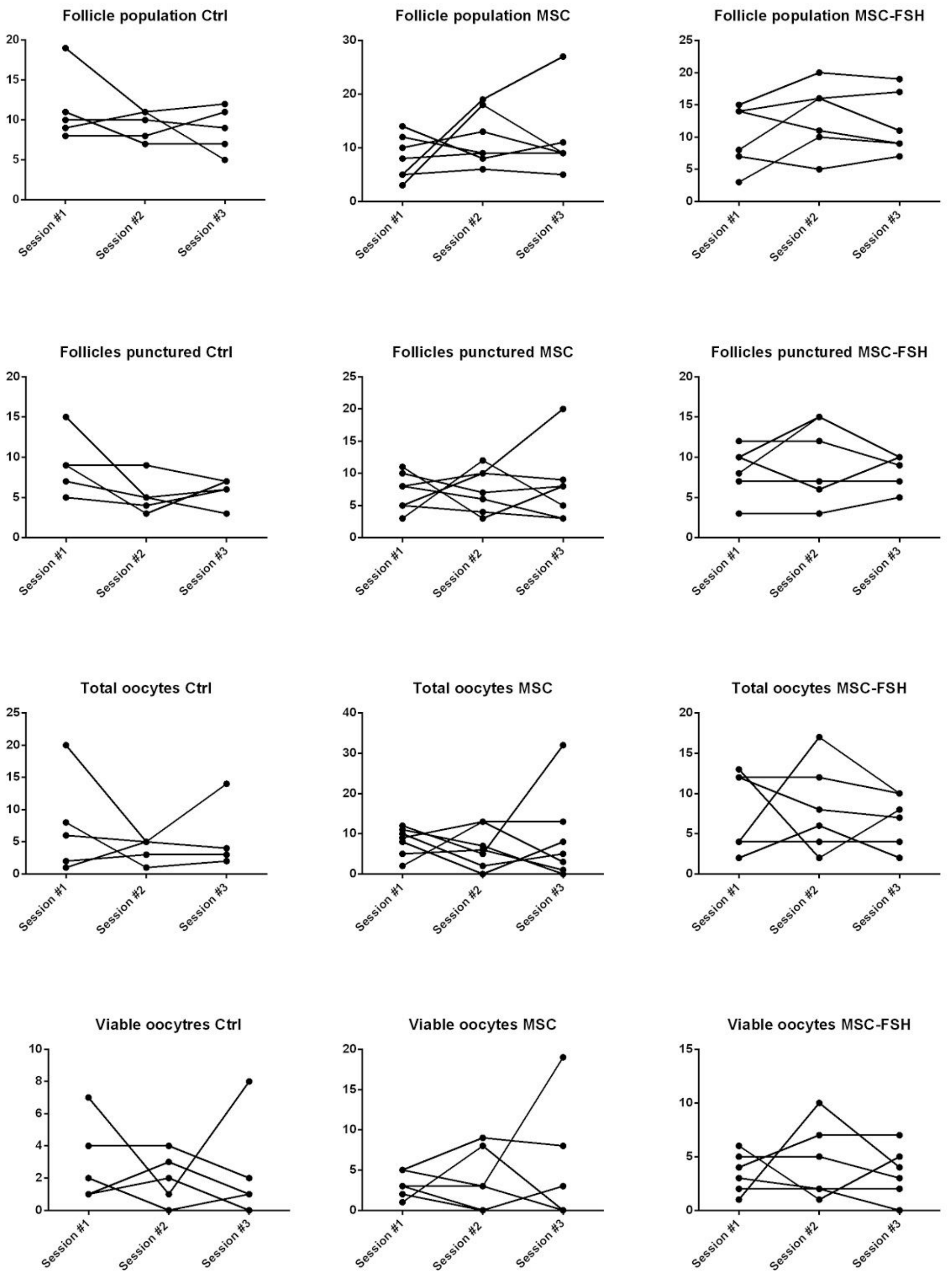

Figure S1. Individual ovarian and OPU outcomes in Gir cows with low IVEP records associated to their use as oocyte donors for long periods (chronic lesion). Cows received PBS (control group), mesenchymal stem cells (MSC group), or mesenchymal stem cells followed by FSH priming (MSC+FSH group). There was no statistical difference among groups (P>0.05).
